# Improved stove interventions to reduce household air pollution in low and middle income countries: a descriptive systematic review

**DOI:** 10.1186/s12889-015-2024-7

**Published:** 2015-07-14

**Authors:** Emma Thomas, Kremlin Wickramasinghe, Shanthi Mendis, Nia Roberts, Charlie Foster

**Affiliations:** British Heart Foundation Centre on Population Approaches for Non-Communicable Disease Prevention, Nuffield Department of Population Health, University of Oxford, Oxford, UK; Chronic Disease Prevention and Management, World Health Organization, Geneva, Switzerland; Bodleian Health Care Libraries, University of Oxford, Oxford, UK

**Keywords:** Improved stoves, Systematic review, Indoor air pollution, Solid fuel smoke, TIDieR checklist

## Abstract

**Background:**

Household air pollution (HAP) resulting from the use of solid fuels presents a major public health hazard. Improved stoves have been offered as a potential tool to reduce exposure to HAP and improve health outcomes. Systematic information on stove interventions is limited.

**Methods:**

We conducted a systematic review of the current evidence of improved stove interventions aimed at reducing HAP in real life settings. An extensive search of ten databases commenced in April 2014. In addition, we searched clinical trial registers and websites for unpublished studies and grey literature. Studies were included if they reported on an improved stove intervention aimed at reducing HAP resulting from solid fuel use in a low or middle-income country.

**Results:**

The review identified 5,243 records. Of these, 258 abstracts and 57 full texts were reviewed and 36 studies identified which met the inclusion criteria. When well-designed, implemented and monitored, stove interventions can have positive effects. However, the impacts are unlikely to reduce pollutant levels to World Health Organization recommended levels. Additionally, many participants in the included studies continued to use traditional stoves either instead of, or in additional to, new improved options.

**Conclusions:**

Current evidence suggests improved stove interventions can reduce exposure to HAP resulting from solid fuel smoke. Studies with longer follow-up periods are required to assess if pollutant reductions reported in the current literature are sustained over time. Adoption of new technologies is challenging and interventions must be tailored to the needs and preferences of the households of interest. Future studies require greater process evaluation to improve knowledge of implementation barriers and facilitators.

**Review registration:**

The review was registered on Prospero (registration number CRD42014009796 ).

**Electronic supplementary material:**

The online version of this article (doi:10.1186/s12889-015-2024-7) contains supplementary material, which is available to authorized users.

## Background

The health impact of indoor air pollution among low-and middle-income countries (LMIC) is considerable and primarily results from solid fuel smoke [1]. Solid fuels such as dung, coal, wood and agricultural residues are used by approximately half of the world’s population for cooking and heating [2, 3]. The burning of these fuels in open fires or inadequate stoves results in harmful pollutants being emitted into the household atmosphere [4].

The 2010 Global Burden of Disease Study ranked household air pollution (HAP) as the third highest global risk factor [5] . In South Asia and sub-Saharan Africa, HAP accounted for the first and second highest risk factor for burden of disease respectively. The study attributed 3.5 million deaths and 4.3 per cent of global disability-adjusted life years (DALYs) in 2010 to HAP from solid fuels [5]. The two major health outcomes associated with HAP are acute lower respiratory infections (ALRI) in children under five years of age and chronic obstructive pulmonary disease (COPD) in adults over 20 years [6]. Women and young children are frequently at greater risk due to longer hours spent indoors [7].

Improved (i.e. high-efficiency and low emission) stoves have been offered as a potential tool to reduce exposure to indoor air pollution, improve health outcomes and decrease greenhouse gas emissions and deforestation [8]. During the 1970s higher oil prices, increasing deforestation and concerns of a “fuelwood crisis” created additional pressure on governments and non-government organisations (NGOs) to act [9]. Many NGOs and governments then facilitated the wide-scale distribution of stoves. Initial enthusiasm about stoves was often supported by laboratory-based experiments performed in highly controlled contexts [9, 10]. The intervention impact in real world contexts was unrealized. Many organisations believed the improved efficiency of the stoves would be enough to facilitate their widespread adoption. However, traditional, “three-stone” biomass stoves have additional benefits such as heating, protection from insects, and wide variety of fuel flexibility [9]. Additionally, improved stoves must be adopted and maintained by households in order to achieve intended benefits. Despite NGO-led practices of stove distribution and improved epidemiological surveillance, what remains unknown are the best ways of implementing improved stoves.

We aimed to conduct a systematic review of stove interventions that aim to reduce household air pollution in LMIC. In-depth understanding of these interventions is required in order to facilitate policy and funding decisions. Additionally, information on the type, quality and distribution of stove interventions is required to facilitate successful replication and scale-up.

## Methods

### Search strategy

Our review was registered on Prospero (registration number CRD42014009796) and followed the Preferred Reporting Items for Systematic Reviews and Meta-Analyses (PRISMA) review process [11] (Additional file 1). In April 2014, we searched the following ten databases: CINAHL (EBSCOHost)[1982-present], Cochrane Central Register of Controlled Trials (Cochrane Library, Wiley)[Issue 4, 2014], Embase (OvidSP)[1974-present], Global Health (OvidSP)[1973-present], Ovid MEDLINE(R) In-Process & Other Non-Indexed Citations and Ovid MEDLINE(R) (OvidSP)[1946-present], PsycINFO (OvidSP)[1967-present], Science Citation Index (Web of Science, Thomson Reuters)[1945-present], Global Health Library – Regional Indexes & WHOLIS http://www.globalhealthlibrary.net/php/index.php and Pubmed http://www.ncbi.nlm.nih.gov/pubmed/. The search imposed no limit on study design or date of publication. The Embase search strategy is provided in Additional file 2 as an example of the search terms used. In addition, we searched clinical trial registers and websites for unpublished studies and grey literature.

### Eligibility criteria

Many studies measure pollutant outcomes over a series of times and as such non-randomised studies (e.g. interrupted time series and before and after studies) are common. Therefore, in order to gain an in-depth understanding of the scope of stove interventions, we included primary intervention studies regardless of study design. Such designs included: individually randomised trials, cluster-randomised trials, controlled before-and-after studies, interrupted time series and project evaluations. Eligible study participants were exposed to HAP from solid fuels such as dung, wood, agricultural residues and coal for cooking and heating; laboratory-based studies were excluded. Interventions were required to take place in LMIC where HAP has the greatest health consequences and systematic information is limited. As such, interventions in high income economies (as per the World Bank [12]) were excluded. Included studies aimed to reduce pollutant emission/exposure through the use of improved stoves. No limit was imposed on the reported outcome as a reduction in any pollutant or health outcome would be important to capture for future studies and scale-up options.

### Study selection

One reviewer removed obviously irrelevant studies and assessed all remaining titles and abstracts for inclusion. A 10 % sample of abstracts were independently assessed by a second reviewer and crosschecked with 90 % agreement reached. The same two reviewers also discussed any ‘unsure’ abstracts. Articles obtained in full text were then reassessed for inclusion.

### Quality assessment

The quality of the included randomised controlled trails (RCTs) was assessed using the Cochrane Collaboration Risk of Bias Tool. The tool is not appropriate for non-RCT designs and as such was limited to RCTs only. The tool covers six domains of bias: selection bias, performance bias, attrition bias, reporting bias and other bias [13]. For each domain a set of criterion determines if the study is at high risk, low risk or unknown risk.

### Synthesis of the literature

The Intervention Description and Replication (TIDieR) Checklist [14] was used as a foundation for the synthesis of the literature. This checklist is an extension of the CONSORT 2010 statement (item 5) and the SPIRIT 2013 statement (item 1) and provides key areas of the intervention that should be reported to enhance replication and implementation of interventions [14]. The TIDieR checklist items include the following elements of the intervention: brief name, why, what, who provided, how, where, when and how much, tailoring, modifications and how well. The TIDieR guide provides an explanation and elaboration of each item [14]. The guidance is intended to apply across all evaluation study designs and reviews.

## Results

**Fig. 1:**
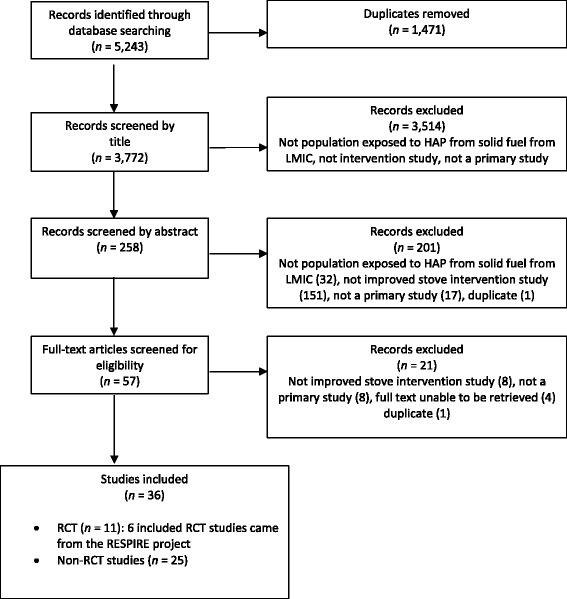
Flow diagram of study selection

Our systematic review identified 5243 potential articles. After duplicates were removed, title screening occurred on 3772 studies of which 258 were further screened on abstract and 57 full texts retrieved. A total of 36 studies were found to meet the full inclusion criteria (see Fig. 1 for a flow chart of study selection). Studies were excluded on the grounds of: study type (only stove intervention or evaluation of stove intervention studies were included); source of air pollution (populations exposed to non-solid fuels only such as tobacco, radon or outdoor sources of air pollution were excluded), the study setting (intervention occurring in non-natural settings such as laboratory-based or non-residential settings such as occupational settings were excluded); the study country (only studies from LMIC were included).

### Reported effect of stove intervention

We examined the effect of stove interventions as reported by study authors. The majority of authors reported a positive reduction of HAP after the installation of an improved stove. Primarily, the main pollutants measured were carbon monoxide (CO) and particular matter (PM). The time frames of measuring pollutant concentration differed greatly across each study from hourly to seven-day measurements. A meta-analysis of the results was not possible due the disparity between pollutant types, methods, and timing of measurement. While pollutant reductions were reported, frequently, these reductions were not enough to meet WHO air quality recommendations. The study by Hanna et. al. (2012) which has the longest follow-up (4 years) showed no improvement post one year. Beltramo et al’s. [15] solar oven intervention group was *higher* than the control group (8.09 ppm/h compared to 6.50 ppm/h respectively). The authors reported this unexpected increase in CO exposure was largely due to smaller household size in the intervention group.

A wide range of health outcomes were reported across the studies. Self-report measures within studies reported a reduction of respiratory (e.g. cough, phlegm, wheeze, chest tightness) [16, 17], non-respiratory (e.g. eye discomfort, headache, backache) [18, 19] and sleep symptoms (e.g. snoring, nasal congestion) [17, 20] in intervention groups. However, objective measures of pulmonary function were less conclusive. The RESPIRE study did not significantly improve women’s lung function or reduce physician-diagnosed pneumonia for children younger than 18 months after 12–18 months of improved stove use [16, 21]. Authors of the RESPIRE study suggest that stove or fuel interventions with lower average emissions than the *plancha* chimney stove may be required for communities with such high exposure to air pollution [21]. Similarly, Clark et. al. [22] found no evidence of association between stove type and lung function. The RESPIRE study did report a non-statistically significant reduction in low birth weight in the intervention groups [23], evidence of a reduction in blood pressure [24] and reduced occurrence of non-specific ST-segment depression [25] suggesting improved stove interventions may potentially affect cardiovascular health.

### Description of study characteristics using the TIDieR checklist

The TIDieR checklist was completed for each included study. Extracted information included: brief name of the intervention *(Item 1);* the rationale and goal *(Item 2),* the stove type and educational material provided *(Item 3)*; who provided the intervention *(Item 4)*, the mode of delivery *(Item 6)*; where the intervention occurred *(Item 7)*; the intervention schedule (Item 8); whether the intervention was tailored *(Item 9)* or modified during the course of the study *(Item 10)*; and whether intervention adherence and fidelity was assessed *(Item 11 and 12).*

### Item 1 & 2: brief name & why

A brief description of each study can be seen in Tables 1, 2 and 3. Nine studies (6 RCTs, 3 non-RCTs) were affiliated with the Randomized Exposure Study of Pollution Indoors and Respiratory Effects (RESPIRE) study [16, 18, 19, 21, 23–27]. This was the first RCT to investigate the health effects from solid fuel use [19]. The study occurred in Guatemala and aimed to assess the impact of improved stoves (*planchas*) on exposure and health outcomes in a rural population reliant on wood fuel. An additional five RCTs were identified, all of which investigated the impact of improved stoves on either exposure outcomes (*n* = 2) or both exposure and health outcomes (*n* = 3). The majority of identified non-RCTs were pre-post studies investigating the impact on exposure-related health outcomes or exposure reduction of various stove types. Some studies also assessed traditional cooking practices and the acceptability of stoves to the local community members.

**Table 1:** Randomised control trials from the Randomized Exposure Study of Pollution Indoors and Respiratory Effects (RESPIRE) study

First author of study, year	Brief name	Study design	Study country	N	Age of partici-pants (years)	Sex	Control group (Y or N)	Pollutant outcome	Health Outcome	Follow-up period (post stove installation)	Reported effect of stove use^(positive effect (+); negative effect (−); no effect (/))^
Diaz, 2008 [18]	RESPIRE: self-rated health among women in the RESPIRE trial	RCT (subsample)	Guatemala	169 (80 Ix; 89 control)	Adult	Female	Y	NA	Self-report of health	Approx. 18 months	+
Diaz, 2007 [19]	RESPIRE: eye discomfort, headache and back pain	RCT (subsample)	Guatemala	504 (259 Ix; 245 control)	Adult	Female	Y	e-CO	NA	12 – 18 months	+
Smith, 2010 [20]	RESPIRE : trial of woodfire chimney cook stoves	RCT	Guatemala	515 infants; 532 mothers	Infants (0–18 months); mothers (15–55 years)	Female & children	Y	CO	In separate papers	Every 3 months >until the children reached 18 months	+
Smith, 2011 [21]	RESPIRE : effect on childhood pneumonia	RCT	Guatemala	534 households (269 intervention; 265 control)	Infants (0–18 months); mothers (15–55 years)	Female & children	Y	CO	Childhood pneumonia	Every 3 months until the children reached 18 months	+
Smith - Sivertsen, 2009 [22]	RESPIRE : Effect on women’s respiratory symptoms and lung function	RCT	Guatemala	504 women	15-55 years	Female	Y	CO	Chronic respiratory symptoms and lung function	Every 3 months until the children reached 18 months	+
Thompson, 2011 [23]	RESPIRE : impact of reduced maternal exposure on new born birth weigh	RCT (Subgroup of RESPIRE)	Guatemala	174 infants (69 from Ix; 105 from control)	Infant s	Both	Y	CO	Birth weight	Until birth	+

*CO* Carbon Monoxide, *e-CO* exhaled CO, *Ix* intervention

**Table 2:** Additional randomised control trials (non-RESPIRE studies)

First author of study, year	Brief name	Study design	Study country	N	Age of partici-pants (years)	Sex	Control group (Y or N)	Pollutant outcome	Health Outcome	Follow-up period (post stove installation	Reported effect of stove use^(positive effect (+); negative effect (−); no effect (/))^
Beltramo 2012 [15]	Provision of solar oven + training + education	RCT	Senegal	790 participants (465 Ix; 325 control)	Mean 23 years	Female	Y	CO	NA	6 months	/
Hanna, 2012 [8]	Household behaviour on the impact of improved cook stoves	RCT (stepped wedge)	India	2651 house-holds	Unknown	Female	Y	e-CO, proxy PM	Exposure-related health complaints and health checks	4 years	/after first year
Jary, 2014 [31]	Feasibility of RCT of cook stove interventions	Pilot parallel RCT	Malawi	50	Adults	Female	Y	e-CO	Symptom burden, oxygen saturation	7 days	Feasible
Romieu, 2009 [36]	Improved biomass stove intervention in rural Mexico	RCT	Mexico	552 women	Adult	Women	Y	CO, PAH	Respiratory & lung function measurements, blood samples & health questionnaire	10 months	+
Rosa, 2014 [35]	Impact of water filters and improved cook stoves on drinking water and HAP	RCT (parallel household – randomised RCT)	Rwanda	566 households (HAP sampling in 121 households)	All	Both	Y	PM2.5	NA	5 months	+

*CO* Carbon Monoxide, *e-CO* Exhaled CO, *PM* particular matter, *PAH* polycyclic aromatic hydrocarbons, *Ix* intervention group

**Table 3:** Non-randomised controlled trials included in review

First author of study, year	Brief name	Study design	Study country	N	Age of participants (years)	Sex	Control group (Y or N)	Pollutant outcome	Health outcome	Follow-up period (post stove installation)	Reported effect of stove use^a^
Before and after studies											
Accinelli, 2014 [17]	Impact of biomass fuel stoves on respiratory and sleep symptoms in children	Before-and-after study	Peru	82	<15	Both	N	NA	Respiratory & sleep symptoms	2 years	+ when exclusive use of stove
Castaneda, 2013 [20]	Effect of improved stoves on sleep apnoea in childre	Before-and-after study	Peru	59	<15	Both	N	NA	Sleep symptoms	1 year	+
Clark, 2013 [28]	Impact of cleaner stoves on blood pressure	Before-and-after study	Nicaragua	74	Adults	Female	N	CO, PM2.5	Blood pressure	9 months – 1 year	+
Cynthia, 2008 [40]	Reduction of PM and CO as a result of the Patsari cookstove	Before-and-after study	Mexico	60 households	Adult	Female	N	CO, PM2.5	NA	1 month	+
Fitzgerald, 2012 [41]	Cookstove interventions in Peru	Before-and-after comparative study	Peru	57 house-holds:30 (stove 1); 27 (stove 2)	18 – 45	Female	N	CO, PM2.5	NA	3 weeks	+
Li, 2011 [29]	Exposure reduction of stove intervention	Before-and-after comparative study	Peru	Program A) 30; Program B) 27 house-holds	18-45	Female	N	CO, PM2.5	Urinary OH-PAH levels	3 weeks	+
Mukhopadhyay 2012 [34]	Exploratory study of cookstoves to inform large-scale interventions	Before-and-after feasibility study	India	32 house-holds	All	Both: focus on primary cooks	N	CO, PM2.5	NA	12 weeks	NA
Oluwole, 2013 [42]	Effect of stoves on HAP and respiratory health in Nigeria	Before-and-after pilot study	Nigeria	59 mother-child pairs	Mother (20–60); child (6–17)	Female & children	N	CO, PM2.5	Exposure-related health complaints	I year	+
Pennise, 2009 [43]	Air quality of improved stoves in Ghana and ethanol stove in Ethiopia	Before-and-after comparative study	Ghana and Ethiopia	Ghana: 36 households; Ethiopia 33 households	All	Both	N	CO, PM2.5	NA	Unclear	+
Riojas-Rodriguez [44]	Impact of Patsari improved stoves on PAHs and CO (subproject of Romieu et al’s RCT)	Before-and-after study	Mexico	63 women	Adult	Women	Y	CO, PAH	Measured in Romieu’s 2010 study	10 months	+
Singh, 2012 [33]	Mud improved stove in Nepal	Before-and-after study	Nepal	47 households	All	Primary cooks (mainly female)	N	CO, PM	Exposure-related health questionnaire	3 & 12 months	+
Torres-Dorsal, 2008 [32]	Evaluation of risk reduction program using biomarkers of exposure and effect	Before-and-after study	Mexico	20 participants	Children (5–17); adult (20–35)	Both	N	COHb	Urinary 1-OHP levels and DNA damage	Unknown	+
Zuk, 2007 [45]	Impact of improved wood stoves in rural Mexico	Before-and-after study	Mexico	53 households	All	Both	N	PM2.5		2-3 months	+
Cross-sectional study											
Bruce, 2004 [46]	Impact of improved stoves, house construction & child location on IAP levels	Cross-sectional	Guatemala	204 house-hold	<1.5	Both	Y	CO, PM3.5	NA	2-3 years	+
Clark, 2009 [22]	Impact of improved stoves on IAP and health	Cross-sectional	Honduras	79	Adult	Female	Y	CO, PM2.5	Pulmonary function, respiratory symptoms, CRP concentrations	NA	+
Guarnieri, 2014 [27]	RESPIRE: airway inflammation	Cross-sectional (within RCT)	Guatemala	45 (19 Ix; 26 control)	Adult	Female	Y	CO, e-CO	Spirometry & induced sputum for cell counts, gene expressions & protein concentrations	18 – 24 months	+
Hartinger, 2013 [47]	Chimney stoves compared to traditional open stoves	Cross-sectional (within RCT)	Peru	93 house-holds (43 Ix; 48	All	Both	Y	CO, PM2.5	NA	7 months	/unless restricted to full
Henkle, 2010 [48]	Honduras stove project	Cross-sectional	Honduras	34 homes	2-84	Both: female (56.4 %)	N	CO, TSP	Respiratory surveys, PEFR	NA	Feasible
Cohort study											
Chapman, 2005 [49]	Improved stoves impact on COPD	Retrospective cohort study	China	20,453	Born 1917-51	Focus on farmers	Y	NA	COPD diagnosis	Average 12.8 years	+
Marketing and campaign											
Joint UNDP [30]	Energy Sector Management Assistance Program (ESMAP) : Niger improved stoves project	Marketing and campaign	Niger	40,000 stoves sold	All	Focus on women	N	NA	NA	NA	Successful marketing and sale of stoves
Mixed study design											
McCracken, 2011 [25]	RESPIRE: effect on ST-segment depression on ECG	Before-and-after study & between group comparative study (RCT subsample)	Guatemala	119 (49 Ix; 70 control)	38 – 48	Female	Y	PM2.5	HRV & ST-segment values	3 weeks	+
McCracken, 2007 [24]	RESPIRE: effect of blood pressure	Before-and-after study & between group comparative study (RCT subsample)	Guatemala	119 (49 Ix; 70 control)	38 – 48	Female	Y	PM2.5	BP	~300 days	+
Non-randomised controlled trial											
Albalak, 2001 [50]	PM reductions of improved cook stoves and LPG fuel use	Non-randomised controlled trial	Guatemala	30 house-holds	All	Both	Y	PM3.5	NA	6 months	+
Baris, 2007 [51]	A multisectoral intervention program in rural China	Non-randomised controlled trial	China	5500 house-holds	All	Both	Y	RPM, CO, SO2	Exposure-related health complaints (e.g., dyspnea, nasal mucous)	12 months	+ when heating main energy source
Zhou, 2006 [52]	Community effectiveness of stove and health education in China (same project as Baris,2007)	Community-based non-randomised controlled trial	China	5500 households	All	Both	Y	RPM, CO & SO2	Selected health indicators for women and children	12 months	+ when heating main energy source

*CO* carbon monoxide, *PM* particulate matter, *PAH* polycyclic aromatic hydrocarbons, *Urinary OH-PAH* urinary hyroxylated PAH, *COHb* carboxyhemoglobin, *e-CO* exhaled carbon monoxide, *TSP* total suspended particulate, *PEFR* peak expiratory flow rate, *COPD* chronic obstructive pulmonary disease, *HRV* heart rate variability, *BP* blood pressure, *SO2* sulphur dioxide, *RPM* respirable particulate matter

^**a**^positive effect (+); negative effect (−); no effect (/)

Additional file

### Item 3 & 4: what (materials & procedures)

Across the studies more than 15 different stove types were used. The most commonly reported stove was the *plancha* (largely due to the RESPIRE study), which is an improved chimney woodstove typically built into the home. Other stoves were portable and delivered to the home (e.g. the Eco-Stove [28]) or provided in multiple pieces and built by the household with provided instructions (e.g. the Juntos National Program [29]). In Hanna et al. [8], households were responsible for providing mud for the stove base, labour and payment of about US$0.75 to pay the mason who assisted in building and maintaining the stove. One study investigated the use of a solar oven stove (the HotPot) [15].

### Item 5: who provided

Studies were largely led by University and NGO collaborations. The most wide-spread dissemination of stoves (*n* = 40,000) was led by the Joint UNDP/ World Bank Energy Sector Management Assistance Program [30]. This program involved training of metalsmith workers, establishment of commercial networks and sensitisation campaigns. Multiple studies recruited bilingual community members or community health workers as field staff. Local brick masons or metalsmiths were frequently used to assist in the building of stoves. Some studies identified community members who used and maintained stoves correctly and employed them as stove inspectors or promoters within communities [8].

### Item 6: how

Table 4 provides a summary of key components of interventions. The majority of studies focused on stove provision only (including education and training on stove use). Very few studies have combined stove provision with additional interventions to reduce HAP such as improving the living environment (e.g., improved kitchen design and ventilation) or modifying user behaviour (e.g., using pot lids, removing children from cooking area).

**Table 4:** Key study components of included studies

Intervention	Articles^a^
Stove provision	29
Comparison of 2 or more stove types	5
Stove + behavioural intervention	3
Stove + changes to home environment	1
Marketing campaign	1
Combined stove + other environmental intervention	1

^a^Some studies fall into 2 categories

### Item 7: where

**Fig. 2:**
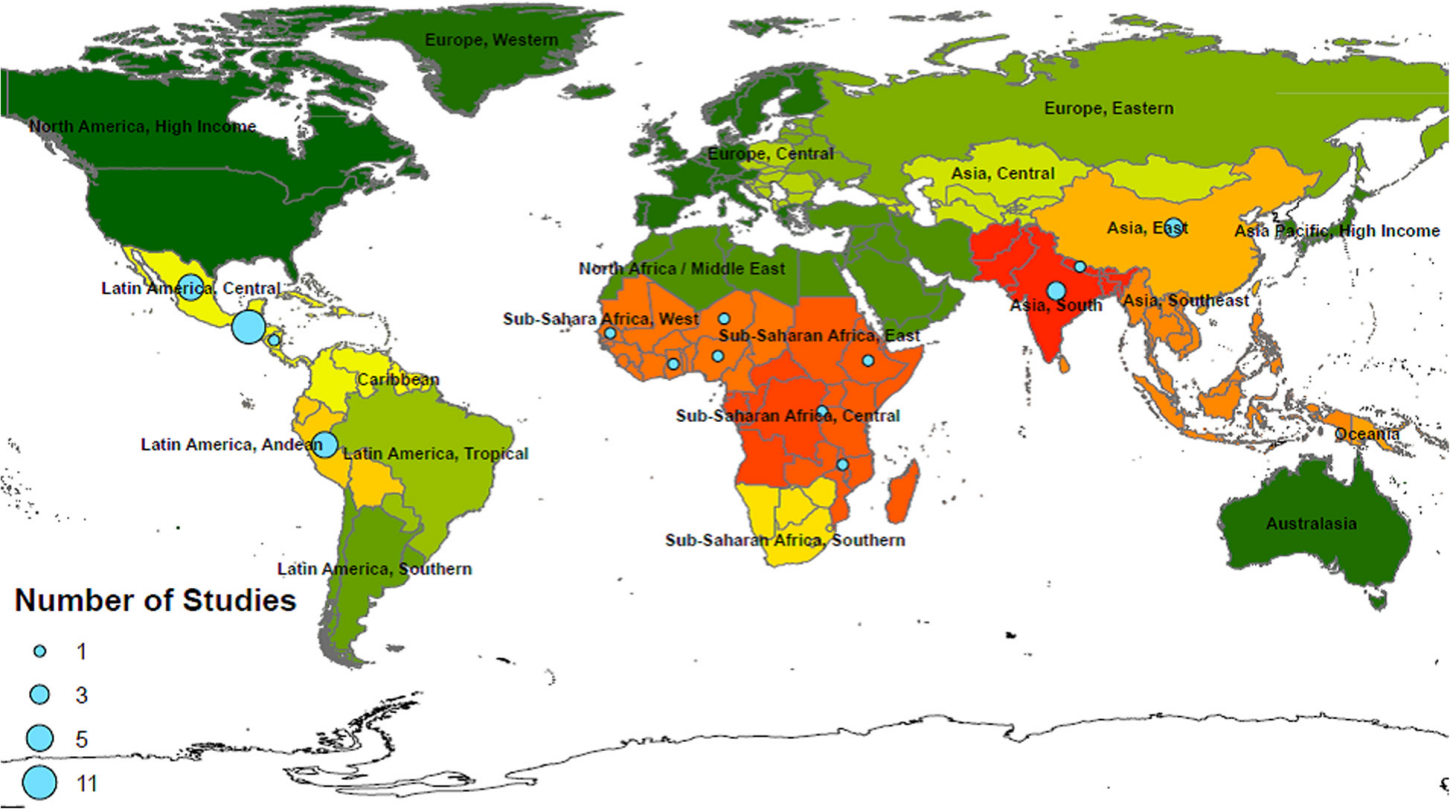
The location of included studies. Countries are grouped as per the Global Burden of Disease regions and colour coordinated in terms of burden of disease attributable to HAP from solid fuels. The numbers of studies in each region are illustrated by the size of the circular marker. High to low burden of disease attributable to HAP from solid fuels as per Lim et. al. [5] is represented by  signifying highest levels of disease burden to  signifying lowest levels of disease burden. This figure was created by the authors using ArcInfo 10.2.1

Figure 2 shows the distribution of study countries identified in the review. In-keeping with the inclusion criteria, only studies from LMIC were included. Fifteen different study countries were identified across Central and South America, Africa and Asia. Figure 2 groups study countries according to the Global Burden of Disease Regions. The countries are colour-coded to highlight areas of high burden of disease attributable to HAP from solid fuels as per Lim et.al. ^5]^. Studies have occurred across a range of settings and locations. Importantly, a range of studies have occurred in South Asia and Sub-Saharan Africa where HAP from solid fuels has the highest disease burden.

### Item 8: when and how much

Most commonly, studies performed baseline assessments and then provided (or installed) the stove with instructions on stove use. Limited interaction between stove installation and post-intervention follow-up was reported. If stoves were damaged or malfunctioning, participant were often responsible for contacting an appropriate person. Uniquely, the RESPIRE study provided weekly maintenance checks.

Post-intervention follow-up ranged from seven days [31] to four years [8]. In Hanna et. al.’s [8] study no meaningful reduction of HAP was seen beyond the first year. No other studies provided follow-up periods beyond two years.

### Item 9 & 10: tailoring & modifications

The majority of large-scale and RCT studies included preliminary questionnaires and needs assessments to determine baseline information and cooking practices. Impressions and observed reactions about the improved stoves were reported to impact upon design and dissemination of some interventions [26, 30]. However, once the intervention was designed, limited tailoring took place across studies. In-built stoves were designed around the house requirements and occasionally adjustments were individualised to each study house [32]. Mostly, however, stove provision was standardised across households and limited adaption took place. In one study, abnormally sized or shaped kitchens were excluded [33].

### Item 11 & 12: how well (adherence & fidelity)

Many studies reported difficulties with adherence and adoption of stoves. ‘Stacking’, the use of traditional stoves in conjunction with improved stoves, was a frequently reported issue. However, while commonly reported as a potential issue, few studies (6/36) actively measured and reported adherence or fidelity data. One study objectively measured stove use with the Stove Use Monitoring System (SUMS) [34] which is fixed to the stove and records the temperature profile over time. Random spot checks by Rosa et. al. [35] and Romieu et. al. [36] reported 64.1 % and 50 % of checked households to be exclusively using the improved stoves. Romieu et. al. [36] conducted a predictive model with longitudinal data to assess factors influencing adoption. The authors reported no clear effect of socio-economic status or education level on stove adoption. Importantly, previous use of a similar stove type was a predictor of improved stove adoption and as such, the authors concluded that greater reinforcement and training of stove use is required in future studies [36]. Much higher adoption rates were reported by the Joint UNDP/ World Bank Energy Sector Management Assistance Program in Niger [30]. This program reported that 96 % of consumers in inspected homes were using the stoves properly, 76 % maintaining it correctly and 93 % had decided to replace it when necessary [30]. This large scale program utilised a sensitisation campaign and publicity to inform both women and men about the existence and advantages of the stove as well as create a market demand. Training on stove use was also provided. Additionally, the design of both the campaign and stove were piloted and modified based on contextual needs.

### Study quality

**Fig. 3:**
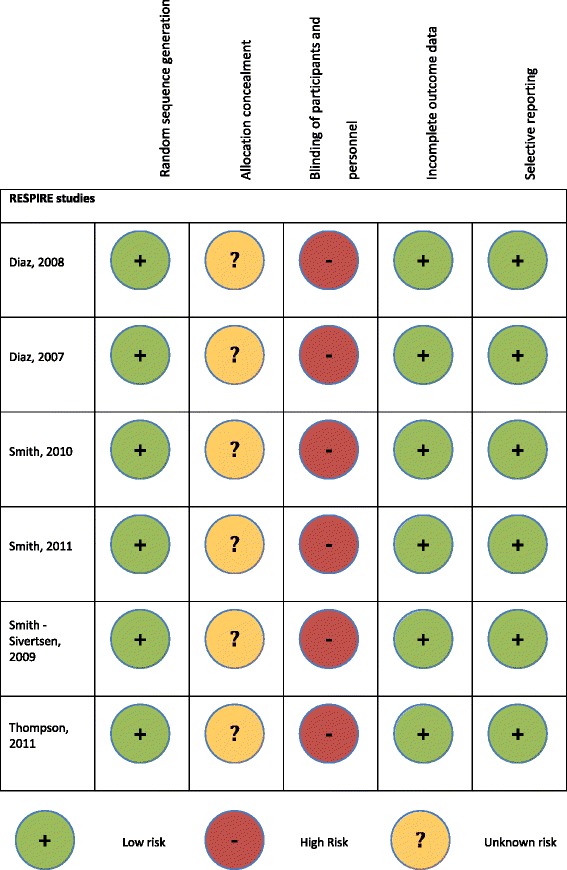
Quality Assessment using the Cochrane Collaboration Risk of Bias Tool of the RESPIRE studies

**Fig. 4:**
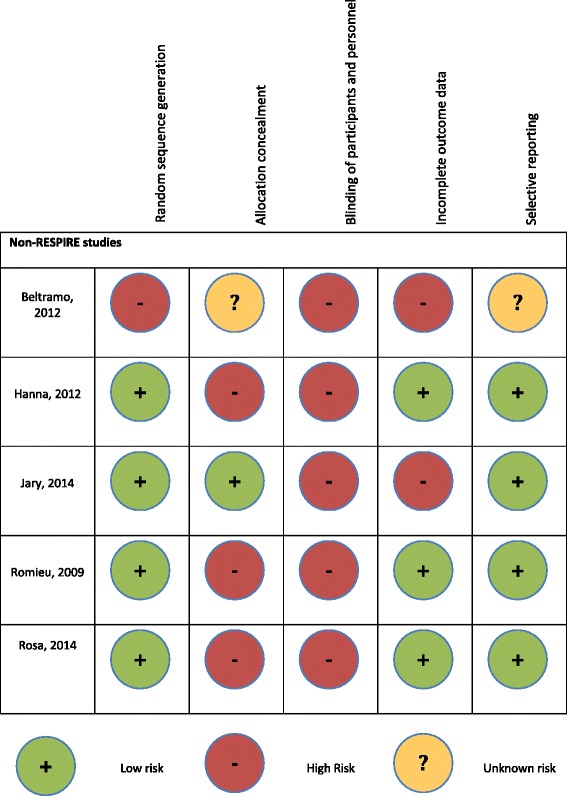
Quality Assessment using the Cochrane Collaboration Risk of Bias Tool of the non-RESPIRE studies

To date, the RESPIRE studies (Fig. 3) have the highest study quality as per the Cochrane Collaboration Risk of Bias Tool. Great variation exists between the additional RCTs (Fig. 4) with no study achieving all of the study criteria.

## Discussion

### Findings and comparison to the literature

We found evidence of well designed and implemented interventions. We were not able to make firm conclusions on the relative impact of the intervention due to the differences in their outcome measures. Potentially, the impact of the interventions may decrease over time. Findings from one study with a 4-year follow-up period reported a significant drop in HAP reduction beyond one year. However, the majority of studies have follow-up periods less than 18 months. Success of stove interventions are heavily dependent on how well households adopt the intervention and exclusively use the improved stove above traditional options. These difficulties are echoed in Barnes et al.’s 1994 comparative review of international stove programs which concluded that “no matter how efficient or cheap the stove, individual households have proved reluctant to adopt it if it is difficult to install and maintain or less convenient and less adaptable to local preferences than its traditional counterpart” [9]. Sensitisation campaigns and publicity such as that used by the Joint UNDP/World Bank in Niger may enhance adoption [30]. Without an in-depth understanding of contextual drivers for implementation success, interventions aimed at reducing HAP cannot be expected to succeed.

### Comparison to WHO air quality targets

We found a disconnection between the relative impact of studies and the targets in the WHO Air Quality Guideline [37]. Pollutant concentrations were measured over different time frames for each included study varying from hourly to 7-day measurements. In general, these did not align to timeframes used in WHO guidelines making comparisons problematic. The WHO targets also appear unachievable for some contexts given the exceptionally high baseline pollutant concentrations. For example, the study by Rosa et. al. [35] report the mean PM^2.5^ concentrations as 48 % lower in the intervention group than the control. However, the levels remained more than six times higher than the WHO interim target for PM_2.5_ (485 μg/m^3^ compared to 75 μg/m^3^ respectively). The authors also noted that even the outdoor cooking areas had concentration levels well above recommended targets (243 μg/m^3^ compared to 75 μg/m^3^ respectively). Given the extreme levels of HAP reported in some studies, single interventions (e.g. provision of a stove) are unlikely to independently reduce pollution to meet recommended standards. As such, complex interventions involving multiple components may be required, however, the literature in this area remains scarce.

### Strengths and weaknesses of the review

The review was strengthened by a wide search across multiple databases. Additionally, the inclusion of a wide variety of study designs enabled the vast contribution of non-randomised study in this field to be included. Further, all studies were carried out in ‘real life’ contexts in communities who regularly use solid fuels for cooking and heating. The wide variety in included studies interventions and outcome measures made comparison between studies difficult.

### Implications for policy and practice and future research

Stove interventions can reduce exposure to HAP. However, we have very little information on *how* these approaches can be implemented in a sustainable way to enhance long-term use. We also lack information on how interventions can be scaled up and what supporting structures are required to assist in their success. As repeatedly advocated throughout the literature, there is a need for practice-based evidence of adoption [38]. We suggest future research evaluate and report the process of implementation along with outcome evaluation to enhance the knowledge of how interventions are carried out. Additionally, use of alternative study designs such as natural experimental studies may assist in understanding the population-level impact of large scale stove interventions [39]. In the interim, implementers should ensure an in-depth understanding of the needs and preferences of consumers and the social, financial and environmental context in which they live. Only through active engagement and involvement of the targeted communities can interventions be adequately tailored to meet their needs and be expected to succeed.

## Conclusion

When well designed, implemented and monitored, current evidence suggests stove interventions can reduce HAP. However, the intervention impacts are unlikely to reduce pollutant levels to WHO recommended levels. Studies have reported a significant reduction in exposure-related health complaints. However, objective measures of lung function have not shown statistically significant improvements in stove interventions groups. Studies with longer follow-up periods are required to assess if pollutant reductions reported in the current literature are sustained over time. Adoption of new technologies is challenging and interventions must be tailored to the needs and preferences of the households of interest and must repeatedly reinforce stove use and benefits. We suggest that future studies give greater emphasis to process evaluation and consider natural experimental designs to increase understanding of population-level impact of stove interventions in real world contexts.

## Additional files

Additional file 1:
**PRISMA 2009 Checklist.**
Additional file 2:
**Embase search terms.**

## Abbreviations

ALRI: Acute lower respiratory infections
CO: Carbon monoxide
COPD: Chronic obstructive pulmonary disease
DALYs: Disability-adjusted life years
HAP: Household air pollution
LMIC: Low and middle-income countries
NGOs: Non-government organisations
PM: Particulate matter
RCTs: Randomised controlled trails
TIDieR Checklist: The Intervention Description and Replication Checklist
WHO: World Health Organization.

## References

[1] Fullerton DG, Bruce N, Gordon SB (2008). Indoor air pollution from biomass fuel smoke is a major health concern in the developing world. Trans R Soc Trop Med Hyg.

[2] Kim KH, Jahan SA, Kabir E (2011). A review of diseases associated with household air pollution due to the use of biomass fuels. J Hazard Mater.

[3] Smith K, Mehta S, Maeusezahl Feuz M (2004). Indoor air pollution from household use of solid fuels, in Comparative quantification of health risks: global and regional burden of disease due to selected major risk factors. Trans R Soc Trop Med Hyg.

[4] Eisner MD, Balmes JR, Mason RJ, Broaddus VC, Martin T, King T, Schraufnagel D, Murray JF, Nadel JA (2010). Indoor and Outdoor Air Pollution. Murray and Nadel’s Textbook of Respiratory Medicine: 2-Volume Set.

[5] Lim SS, Vos T, Flaxman AD, Danaei G, Shibuya K, Adair-Rohani H (2012). A comparative risk assessment of burden of disese and injury attributable to 67 risk factors and risk factor clusters in 21 regions, 1990–2010 : a systematic analysis for the Global Burden of Disease Study 2010. Lancet.

[6] Mehta S, Shahpar C (2004). The health benefits of interventions to reduce indoor air pollution from solid fuel use: a cost-effectiveness analysis. Energy Sustain Dev.

[7] Bruce N, Rehfuess E, Mehta S, Hutton G, Smith K, Jamison DT, Breman JG, Measham AR, Alleyne G, Claeson M, Evans DB, Jha P, Mills A, Musgrove P (2006). Indoor Air Pollution. Disease Control Priorities in Developing Countries.

[8] Hanna R, Greenstone M. Up in smoke: the influence of household behavior on the long-run impact of improved cooking stoves. Natl Bur Econ Res. 2012. http://papers.ssrn.com/sol3/papers.cfm?abstract_id=2039004

[9] Barnes DF (1994). What makes people cook with improved biomass stoves?: a comparative international review of stove programs.

[10] Smith KR, Khalil MAK, Rasmussen RA, Thorneloe SA, Manegdeg F, Apte M (1993). Greenhouse gases from biomass and fossil fuel stoves in developing countries: a Manila pilot study. Chemosphere.

[11] Moher D, Liberati A, Tetzlaff J, Altman D, The PRISMA Group (2009). Preferred reporting items for systematic reviews and meta-analyses: the PRISMA statement. PloS Med.

[12] Country and lending groups. The World Bank.(2015). Country and Lending Groups. Accessed online. http://data.worldbank.org/about/country-and-lending-groups

[13] Higgins JP, Altman DG, Gotzsche PC, Juni P, Moher D, Oxman AD (2011). The Cochrane Collaboration’s tool for assessing risk of bias in randomised trials. BMJ.

[14] Hoffmann TC, Glasziou PP, Boutron I, Milne R, Perera R, Moher D (2014). Better reporting of interventions: template for intervention description and replication (TIDieR) checklist and guide. BMJ.

[15] Beltramo T, Levine DI (2013). The effect of solar ovens on fuel use, emissions and health: results from a randomised controlled trial. J Dev Effectiv.

[16] Smith-Sivertsen T, Diaz E, Pope D, Lie RT, Diaz A, McCracken J (2009). Effect of reducing indoor air pollution on women’s respiratory symptoms and lung function: the RESPIRE Randomized Trial, Guatemala. Am J Epidemiol.

[17] Accinelli RA, Llanos O, Lopez LM, Pino MI, Bravo YA, Salinas V (2014). Adherence to reduced-polluting biomass fuel stoves improves respiratory and sleep symptoms in children. BMC Pediatrics.

[18] Diaz E, Bruce N, Pope D, Diaz A, Smith KR, Smith-Sivertsen T (2008). Self-rated health among Mayan women participating in a randomised intervention trial reducing indoor air pollution in Guatemala. BMC Int Health Hum Rights.

[19] Diaz E, Smith-Sivertsen T, Pope D, Lie RT, Diaz A, McCracken J (2007). Eye discomfort, headache and back pain among Mayan Guatemalan women taking part in a randomised stove intervention trial. J Epidemiol Commun Health.

[20] Castaneda JL, Kheirandish-Gozal L, Gozal D, Accinelli RA, Pampa Cangallo Instituto de Investigaciones de la Altura Research G (2013). Effect of reductions in biomass fuel exposure on symptoms of sleep apnea in children living in the peruvian andes: a preliminary field study. Pediatr Pulmonol.

[21] Smith KR, McCracken JP, Weber MW, Hubbard A, Jenny A, Thompson LM (2011). Effect of reduction in household air pollution on childhood pneumonia in Guatemala (RESPIRE): a randomised controlled trial. Lancet.

[22] Clark ML, Peel JL, Burch JB, Nelson TL, Robinson MM, Conway S (2009). Impact of improved cookstoves on indoor air pollution and adverse health effects among Honduran women. Int J Environ Health Res.

[23] Thompson LM, Bruce N, Eskenazi B, Diaz A, Pope D, Smith KR (2011). Impact of reduced maternal exposures to wood smoke from an introduced chimney stove on newborn birth weight in rural Guatemala. Environ Health Perspect.

[24] McCracken JP, Smith KR, Diaz A, Mittleman MA, Schwartz J (2007). Chimney stove intervention to reduce long-term wood smoke exposure lowers blood pressure among Guatemalan women. Environ Health Perspect.

[25] McCracken J, Smith KR, Stone P, Diaz A, Arana B, Schwartz J (2011). Intervention to lower household wood smoke exposure in Guatemala reduces ST-segment depression on electrocardiograms. Environ Health Perspect.

[26] Smith KR, McCracken JP, Thompson L, Edwards R, Shields KN, Canuz E (2010). Personal child and mother carbon monoxide exposures and kitchen levels: methods and results from a randomized trial of woodfired chimney cookstoves in Guatemala (RESPIRE). J Expo Sci Environ Epidemiol.

[27] Guarnieri MJ, Diaz JV, Basu C, Diaz A, Pope D, Smith KR (2014). Effects of woodsmoke exposure on airway inflammation in rural guatemalan women. PLoS ONE [Electronic Resource].

[28] Clark ML, Bachand AM, Heiderscheidt JM, Yoder SA, Luna B, Volckens J (2013). Impact of a cleaner-burning cookstove intervention on blood pressure in Nicaraguan women. Indoor Air.

[29] Li Z, Sjodin A, Romanoff LC, Horton K, Fitzgerald CL, Eppler A (2011). Evaluation of exposure reduction to indoor air pollution in stove intervention projects in Peru by urinary biomonitoring of polycyclic aromatic hydrocarbon metabolites. Environ Int.

[30] Program JUWBESMA (1987). Niger: Improved stoves project.

[31] Jary HR, Kachidiku J, Banda H, Kapanga M, Doyle JV, Banda E (2014). Feasibility of conducting a randomised controlled trial of a cookstove intervention in rural Malawi. Int J Tuberc Lung Dis.

[32] Torres-Dosal A, Perez-Maldonado IN, Jasso-Pineda Y, Martinez Salinas RI, Alegria-Torres JA, Diaz-Barriga F (2008). Indoor air pollution in a Mexican indigenous community: evaluation of risk reduction program using biomarkers of exposure and effect. Sci Total Environ.

[33] Singh A, Tuladhar B, Bajracharya K, Pillarisetti A (2012). Assessment of effectiveness of improved cook stoves in reducing indoor air pollution and improving health in Nepal. Energy Sustain Dev.

[34] Mukhopadhyay R, Sambandam S, Pillarisetti A, Jack D, Mukhopadhyay K, Balakrishnan K (2012). Cooking practices, air quality, and the acceptability of advanced cookstoves in Haryana, India: an exploratory study to inform large-scale interventions. Glob Health Action.

[35] Rosa G, Majorin F, Boisson S, Barstow C, Johnson M, Kirby M (2014). Assessing the impact of water filters and improved cook stoves on drinking water quality and household air pollution: a randomised controlled trial in rwanda. PLoS ONE [Electronic Resource].

[36] Romieu I, Riojas-Rodriguez H, Marron-Mares AT, Schilmann A, Perez-Padilla R, Masera O (2009). Improved biomass stove intervention in rural Mexico: impact on the respiratory health of women. Am J Respir Crit Care Med.

[37] World Health Organization (2006). WHO Air quality guidelines for partinculate matter, ozone, nitrogen dioxide and sulfur dioxide. Global update 2005.

[38] Lewis JJ, Pattanayak SK (2012). Who adopts improved fuels and cookstoves? A systematic review. Environ Health Perspect.

[39] Craig P, Cooper C, Gunnell D, Haw S, Lawson K, Macintyre S, et al. Using natural experiments to evaluate population health interventions: new Medical Research Council guidance. J Epidemiol Commun Health. 2012, jech-2011. 10.1136/jech-2011-200375 PMC3796763 22577181

[40] Cynthia AA, Edwards RD, Johnson M, Zuk M, Rojas L, Jimenez RD (2008). Reduction in personal exposures to particulate matter and carbon monoxide as a result of the installation of a Patsari improved cook stove in Michoacan Mexico. Indoor Air.

[41] Fitzgerald C, Aguilar-Villalobos M, Eppler AR, Dorner SC, Rathbun SL, Naeher LP (2012). Testing the effectiveness of two improved cookstove interventions in the Santiago de Chuco Province of Peru. Sci Total Environ.

[42] Oluwole O, Ana GR, Arinola GO, Wiskel T, Falusi AG, Huo DZ (2013). Effect of stove intervention on household air pollution and the respiratory health of women and children in rural Nigeria. Air Qual Atmosphere Health.

[43] Pennise D, Brant S, Agbeve SM, Quaye W, Mengesha F, Tadele W (2009). Indoor air quality impacts of an improved wood stove in Ghana and an ethanol stove in Ethiopia. Energy Sustain Devel.

[44] Riojas-Rodriguez H, Schilmann A, Marron-Mares AT, Masera O, Li Z, Romanoff L (2011). Impact of the improved patsari biomass stove on urinary polycyclic aromatic hydrocarbon biomarkers and carbon monoxide exposures in rural Mexican women. Environ Health Perspect.

[45] Zuk M, Rojas L, Blanco S, Serrano P, Cruz J, Angeles F (2007). The impact of improved wood-burning stoves on fine particulate matter concentrations in rural Mexican homes. J Expo Sci Environ Epidemiol.

[46] Bruce N, McCracken J, Albalak R, Schei MA, Smith KR, Lopez V (2004). Impact of improved stoves, house construction and child location on levels of indoor air pollution exposure in young Guatemalan children. J Expo Anal Environ Epidemiol.

[47] Hartinger SM, Commodore AA, Hattendorf J, Lanata CF, Gil AI, Verastegui H (2013). Chimney stoves modestly improved Indoor Air Quality measurements compared with traditional open fire stoves: results from a small-scale intervention study in rural Peru. Indoor Air.

[48] Henkle J, Mandzuk C, Emergy E, Schrowe L, Sevilla-Martir J (2010). Global health and international medicine: Honduras Stove Project. Hispanic Health Care Int.

[49] Chapman RS, He X, Blair AE, Lan Q (2005). Improvement in household stoves and risk of chronic obstructive pulmonary disease in Xuanwei, China: retrospective cohort study. BMJ.

[50] Albalak R, Bruce N, McCracken JP, Smith KR, De Gallardo T (2001). Indoor respirable particulate matter concentrations from an open fire, improved cookstove, and LPG/open fire combination in a rural Guatemalan community. Environ Sci Technol.

[51] Baris E, Ezzati M (2007). Household energy, indoor air pollution and health: a multisectoral intervention program in rural China.

[52] Zhou Z, Jin Y, Liu F, Cheng Y, Liu J, Kang J (2006). Community effectiveness of stove and health education interventions for reducing exposure to indoor air pollution from solid fuels in four Chinese provinces. Environ Res Lett.

