# Supplementary material for: Improved stove interventions to reduce household air pollution in low and middle income countries: a descriptive systematic review
Source: BMC Public Health. 2015 Jul 14;15:650. doi: 10.1186/s12889-015-2024-7 (PMC4499941; doi:10.1186/s12889-015-2024-7)
Supplement: Additional file 2: — Embase search terms. [file 12889_2015_2024_MOESM2_ESM.pdf]

**Embase search strategy**

The following search strategy was utilised for the Embase database search. Additional database searches were based on this strategy. Each numbered lined represents a different combination of key words searched. The right hand column displays the number of articles identified for each of the search combinations.

| | | |
|---|---|---|
| 1 | indoor air pollution/ | 10438 |
| 2 | household/ and (wood/ or coal/ or charcoal/ or peat/ or fossil fuel/) | 193 |
| 3 | ((indoor? or domestic or household? or house hold? or residential) adj5 pollut*).ti,ab. | 3419 |
| 4 | ((indoor? or domestic or household? or house hold? or residential) and pollut*).ti. | 1164 |
| 5 | ((indoor? or domestic or household? or house hold? or residential) adj5 emission?).ti,ab. | 804 |
| 6 | ((indoor? or domestic or household? or house hold? or residential) and emission?).ti. | 328 |
| 7 | (((indoor? or domestic or household? or house hold? or residential) adj3 air) and pollut*).ti,ab. | 2715 |
| 8 | (((indoor? or domestic or household? or house hold? or residential) adj3 air) and quality).ti,ab. | 2084 |
| 9 | (((indoor? or domestic or household? or house hold? or residential) adj3 air) and emission?).ti,ab. | 777 |
| 10 | ((indoor? or domestic or household? or house hold? or residential) and air).ti. | 2777 |
| 11 | (((indoor? or domestic or household? or house hold? or residential) adj3 atmospher*) and pollut*).ti,ab. | 71 |
| 12 | (((indoor? or domestic or household? or house hold? or residential) adj3 atmospher*) and quality).ti,ab. | 17 |
| 13 | (((indoor? or domestic or household? or house hold? or residential) adj3 atmospher*) and emission?).ti,ab. | 16 |
| 14 | ((indoor? or domestic or household? or house hold? or residential) and atmospher*).ti. | 94 |
| 15 | ((indoor? or domestic or household? or house hold? or residential) and (burning or fire? or cook*) and (smok* or pollut* or emission? or fume?)).ti,ab. | 1860 |

| | | |
|---|---|---|
| 16 | ((indoor? or domestic or household? or house hold? or residential) and (wood* or coal or dung or biomass or charcoal or solid fuel?) and (smok* or pollut* or emission? or fume?)).ti,ab. | 1921 |
| 17 | ((indoor? or domestic or household? or house hold? or residential) and (wood* or coal or dung or biomass or charcoal or solid fuel?) and (burning or fire? or cook*)).ti,ab. | 1256 |
| 18 | ((indoor? or domestic or household? or house hold? or residential) and open fire*).ti,ab. | 86 |
| 19 | ((indoor? or domestic or household? or house hold? or residential) and ((toxic or dirty) adj2 (fuel? or pollut* or air or emmission? or fume?))).ti,ab. | 94 |
| 20 | ((environment* adj2 exposure?) and (indoor? or domestic or household? or house hold? or residential) and (wood * or coal or dung or biomass or charcoal)).ti,ab. | 61 |
| 21 | ((environment* adj2 exposure?) and (indoor? or domestic or household? or house hold? or residential) and (burning or fire? or cook*)).ti,ab. | 92 |
| 22 | ((indoor? or domestic or household? or house hold? or residential) and (particulate? or pm or pm1* or pm2* or ultrafine particle* or ultra-fine particle* or fine particle* or coarse particle*)).ti,ab. | 3897 |
| 23 | ((indoor? or domestic or household? or house hold? or residential) and (sulphur oxide? or carbon monoxide? or (nitrogen adj2 (oxide? or dioxide)) or polycyclic hydrocarbon? or volatile organic compound? or co or so2 or nox or voc or vocs)).ti,ab. | 6248 |
| 24 | 1 or 2 or 3 or 4 or 5 or 6 or 7 or 8 or 9 or 10 or 11 or 12 or 13 or 14 or 15 or 16 or 17 or 18 or 19 or 20 or 21 or 22 or 23 | 21558 |
| 25 | household/ and (biofuel/ or kerosene/ or liquified natural gas/ or liquified petroleum gas/) | 88 |
| 26 | Cooking/ | 9994 |
| 27 | cook*.ti,ab. | 23029 |
| 28 | stove?.ti,ab. | 2103 |
| 29 | oven?.ti,ab. | 5113 |
| 30 | (kerosene or lpg or liquid petroleum gas or biogas or biofuel* or ethanol or electricity or solar or thermal or briquette? or pellet?).ti,ab. | 284322 |
| 31 | ((low smoke or smokeless or clean* or green* or environment*) adj5 (fuel* or energy or heating or fire?)).ti,ab. | 5721 |

| | | |
|---|---|---|
| 32 | fire/ | 9370 |
| 33 | air conditioning/ or room ventilation/ | 16774 |
| 34 | (ventilat* or filtrat*).ti,ab. | 270748 |
| 35 | ((air or smoke) adj2 (vent? or filter?)).ti,ab. | 1281 |
| 36 | ((air or smoke) adj2 extract*).ti,ab. | 1411 |
| 37 | (chimney? or flue? or hood?).ti,ab. | 7399 |
| 38 | window?.ti,ab. | 60585 |
| 39 | ((kitchen? or domestic or household? or house hold? or residential) adj5 design*).ti,ab. | 1055 |
| 40 | ((behavior? or behaviour?) adj5 (chang* or modif*)).ti,ab. | 47011 |
| 41 | 25 or 26 or 27 or 28 or 29 or 30 or 31 or 32 or 33 or 34 or 35 or 36 or 37 or 38 or 39 or 40 | 712047 |
| 42 | 24 and 41 | 5373 |
| 43 | limit 42 to "reviews (maximizes specificity)" | 31 |
| 44 | randomized controlled trial/ | 342018 |
| 45 | controlled clinical trial/ | 384017 |
| 46 | single blind procedure/ or double blind procedure/ | 131946 |
| 47 | crossover procedure/ | 38491 |
| 48 | random*.tw. | 872452 |
| 49 | placebo*.tw. | 199953 |

| | | |
|---|---|---|
| 50 | ((singl* or doubl*) adj (blind* or mask*)).tw. | 161062 |
| 51 | (crossover or cross over or factorial* or latin square).tw. | 94552 |
| 52 | (assign* or allocat* or volunteer*).tw. | 489527 |
| 53 | 44 or 45 or 46 or 47 or 48 or 49 or 50 or 51 or 52 | 1508671 |
| 54 | 42 and 53 | 391 |
| 55 | (animal$ not human$).sh,hw. | 3726706 |
| 56 | (rat or rats or cow or cows or chicken? or horse or horses or mice or mouse or bovine or animal?).ti. | 1499050 |
| 57 | 55 or 56 | 4089617 |
| 58 | 54 not 57 | 380 |
| 59 | intervention?.ti,ab. | 656883 |
| 60 | (pre-intervention? or preintervention? or "pre intervention?" or post-intervention? or postintervention? or "post intervention?").ti,ab. | 13417 |
| 61 | demonstration project?.ti,ab. | 2300 |
| 62 | (pre-post or "pre test$" or pretest$ or posttest$ or "post test$" or (pre adj5 post)).ti,ab. | 96092 |
| 63 | (pre-workshop or post-workshop or (before adj3 workshop) or (after adj3 workshop)).ti,ab. | 859 |
| 64 | trial.ti. or ((study adj3 aim?) or "our study").ab. | 862216 |
| 65 | (before adj10 (after or during)).ti,ab. | 444222 |
| 66 | (time points adj3 (over or multiple or three or four or five or six or seven or eight or nine or ten or eleven or twelve or month$ or hour? or day? or "more than")).ab. | 12212 |

| | | |
|---|---|---|
| 67 | pilot.ti. | 50108 |
| 68 | (multicentre or multicenter or multi-centre or multi-center).ti. | 39643 |
| 69 | random$.ti,ab. or controlled.ti. | 926034 |
| 70 | *experimental design/ or *pilot study/ or quasi experimental study/ | 7823 |
| 71 | ("quasi-experiment$" or quasiexperiment$ or "quasi random$" or quasirandom$ or "quasi control$" or quasicontrol$ or ((quasi$ or experimental) adj3 (method$ or study or trial or design$))).ti,ab. | 116907 |
| 72 | ("time series" adj2 interrupt$).ti,ab. | 1141 |
| 73 | 59 or 60 or 61 or 62 or 63 or 64 or 65 or 66 or 67 or 68 or 69 or 70 or 71 or 72 | 2678338 |
| 74 | 42 and 73 | 928 |
| 75 | (animal$ not human$).sh,hw. | 3726706 |
| 76 | (rat or rats or cow or cows or chicken? or horse or horses or mice or mouse or bovine or animal?).ti. | 1499050 |
| 77 | 75 or 76 | 4089617 |
| 78 | 74 not (77 or 58) | 607 |
